# The Efficacy and Safety of Cilostazol vs. Aspirin for Secondary Stroke Prevention: A Systematic Review and Meta-Analysis

**DOI:** 10.3389/fneur.2022.814654

**Published:** 2022-02-15

**Authors:** Erqing Chai, Jinhua Chen, Changqing Li, Xue Zhang, Zhiqiang Fan, Shijie Yang, Kaixuan Zhao, Wei Li, Zaixing Xiao, Yichuan Zhang, Futian Tang

**Affiliations:** ^1^Cerebrovascular Disease Center, Gansu Provincial Hospital, Lanzhou, China; ^2^Emergency General Hospital, Beijing, China; ^3^The First School of Clinical Medicine, Lanzhou University, Lanzhou, China; ^4^Intensive Care Unit 1, Gansu Provincial Hospital, Lanzhou, China; ^5^First Clinical Medical College, Gansu University of Chinese Medicine, Lanzhou, China; ^6^School of Clinical Medicine, Ningxia Medical University, Ningxia, China; ^7^Key Laboratory of Gansu Digestive System Tumor, Lanzhou University Second Hospital, Lanzhou, China

**Keywords:** stroke prevention, efficacy and safety, cilostazol, aspirin, systematic review and meta-analysis

## Abstract

**Background:**

Cilostazol is often used in Asia-Pacific countries for stroke prevention. The current systematic review and meta-analysis aimed to evaluate the effectiveness, safety, and adverse outcomes of cilostazol monotherapy compared to aspirin monotherapy for secondary stroke prevention.

**Methods:**

The researchers conducted a comprehensive research in multiple databases (PubMed, Embase, and Cochrane library) of randomized controlled trials from conception to December 2020. The primary efficacy outcome was the occurrence of any stroke, the primary safety outcome was the bleeding risk, and the primary adverse outcome was the rate of headache and dizziness. The Mantel-Haenszel method was used to calculate a random-effects prediction. Cilostazol and aspirin were compared using a pooled risk assessment with 95% CIs.

**Results:**

Six studies involving 5,617 patients were included in this review. Compared with aspirin monotherapy, cilostazol was associated with significantly lower rates of any strokes (RR: 0.67; 95% CI: 0.55–0.82) and significantly lower bleeding rates [risk ratio (RR): 0.53; 95% CI: 0.37–0.74]. However, compared with aspirin monotherapy, cilostazol was associated with significantly higher rates of headache (RR: 1.77; 95% CI: 1.41–2.20) and dizziness (RR: 1.28; 95% CI: 1.08–1.52).

**Conclusions:**

Consistent with previous studies, cilostazol monotherapy is superior to aspirin monotherapy in reducing the rate of any strokes and the bleeding risk after having a stroke. However, the use of cilostazol monotherapy is associated with several adverse life outcomes such as headaches and dizziness.

## Introduction

A stroke has main clinical manifestations of cerebral ischemia and hemorrhagic injury, having a very high mortality and disability rate (1, 2). Antiplatelets are the major therapy for the secondary stroke prevention (3). Aspirin and cilostazol are the most commonly used antiplatelet agents (4). Most patients who have had a stroke are given aspirin (5). According to two major randomized clinical studies of aspirin in acute ischemic stroke, aspirin decreased the risk of early chronic stroke by ~12% at 2–4 weeks (6). However, aspirin-related cerebral hemorrhage is a complication that is currently of concern (5). Cilostazol was reported to be efficacious for the prevention of stroke recurrence (4), which might be related to the various mechanisms, such as anti-platelet aggregation, anti-atherosclerosis, promotion of vascular endothelial recovery, cell apoptosis inhibition, and practical value for the prevention and treatment of ischemic stroke (5, 7, 8). Studies have shown that cilostazol can be used as a drug to treat ischemic strokes and as a preventive drug for recurrence (9). Shinohara et al. (4) reported that the primary endpoint for prevention of secondary stroke occurred at yearly rates of 2.76% in the cilostazol group and 3.71% in the aspirin group (*p* = 0.0357).

The previous meta-analysis primarily focused on comparing the efficacy and safety of cilostazol monotherapy or dual therapy with clopidogrel and aspirin monotherapy (10–12). However, there is no meta-analysis comparing cilostazol monotherapy to aspirin monotherapy as secondary prevention after stroke and in regard to cilostazol's side effects. Therefore, the researchers conducted a systematic review and meta-analysis to evaluate the efficacy and safety of cilostazol monotherapy compared to aspirin therapy. The researchers will further identify the frequency of the adverse side effects caused by these two treatment arms.

## Methods

### Data Sources

The Preferred Reporting Items for Systematic Reviews and Meta-Analyses (PRISMA) checklist was used to perform our meta-analysis based on the Preferred Reporting Elements for Systematic Assessments (13). Searches were conducted in the following electronic databases from conception to December 2020: PubMed, Embase, and the Cochrane Library. The researchers searched with the following headings: “stroke,” “acute ischemic stroke,” “TIA,” “secondary prevention,” “aspirin,” AND “cilostazol.” The gray literature was searched through OpenGrey and Google Scholar. After searches, all relevant citations were saved in a bibliographic reference manager (EndNote, x9 version, Thomson Reuters). Duplicated results were considered only one time. The titles and abstracts that did not adhere to the established eligibility criteria were excluded. The resulting articles were evaluated and judged by their full text. Additional citations were sought from the analysis of the reference list of all the articles previously selected. The selection process was conducted by two examiners (EC and CL) and checked by a third examiner (FT) in cases of disagreements.

### Selection Criteria and Data Extraction

**Figure 1:**
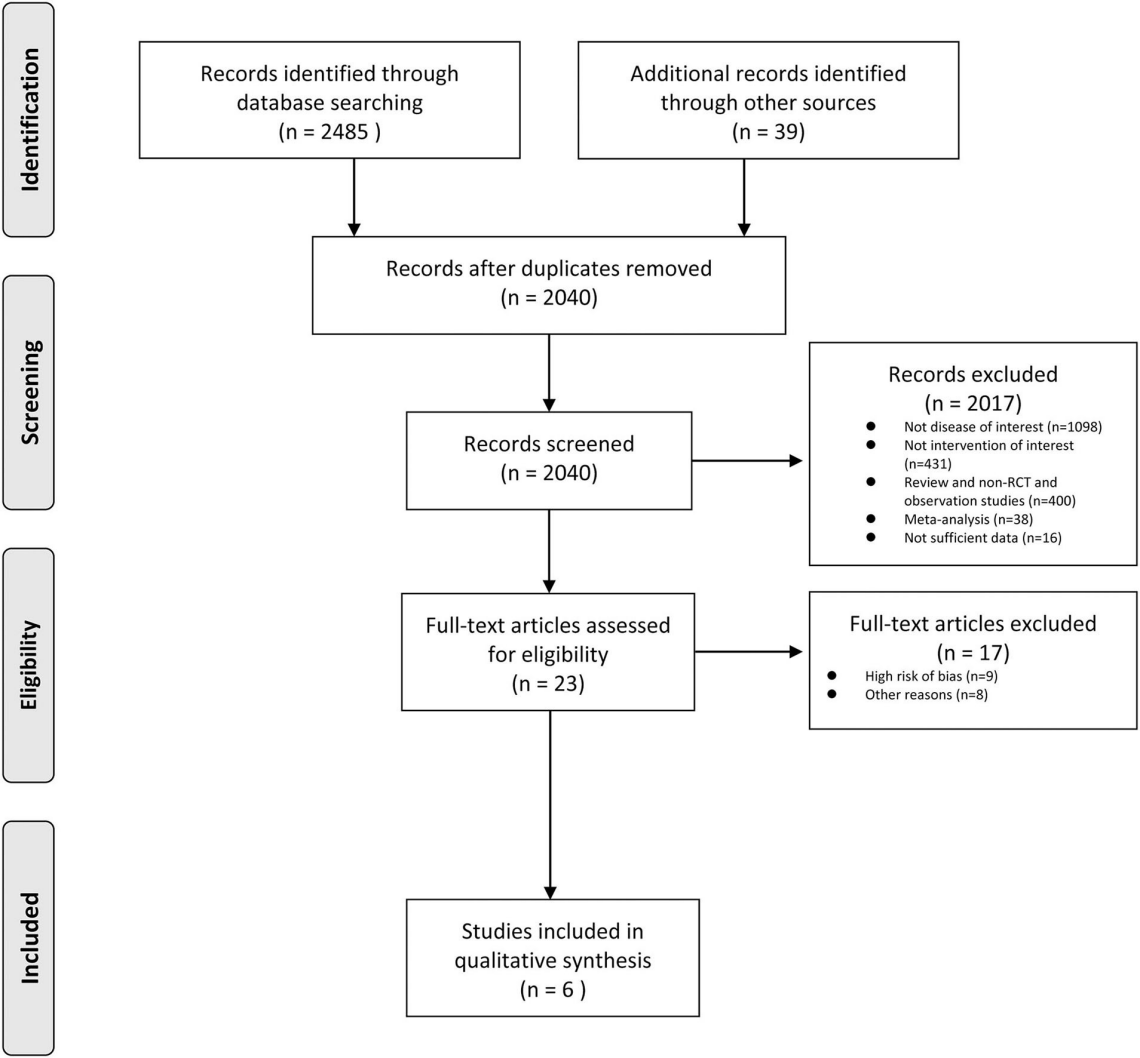
A flowchart.

The inclusion criteria were: (1) randomized controlled studies, (2) a comparison of cilostazol monotherapy with aspirin monotherapy, (3) the efficacy outcomes including recurrent stroke reported, and (4) the adverse outcomes. A total of six studies met the criteria. The exclusion criteria included: (1) non-randomized controlled trials, (2) the cilostazol combination therapy (clopidogrel or aspirin) with an aspirin combination therapy (clopidogrel), (3) only reported efficacy and safety outcomes and no adverse outcomes reported. Two authors independently conducted the research and performed the data extraction (Figure 1).

### Outcomes Measured

The primary efficacy outcome was the occurrence of any stroke (including ischemic stroke and hemorrhagic stroke); the secondary efficacy outcome includes the occurrence of ischemic stroke. The primary safety outcome was intracranial hemorrhage, including subarachnoid hemorrhage and subarachnoid hemorrhage, and other safety outcomes, including bleeding, vascular death, and all-cause mortality. The primary adverse outcome is the headache; the secondary adverse outcome is dizziness where both outcomes include tachycardia and palpitation.

### Assessment of Risk of Bias

**Figure 2:**
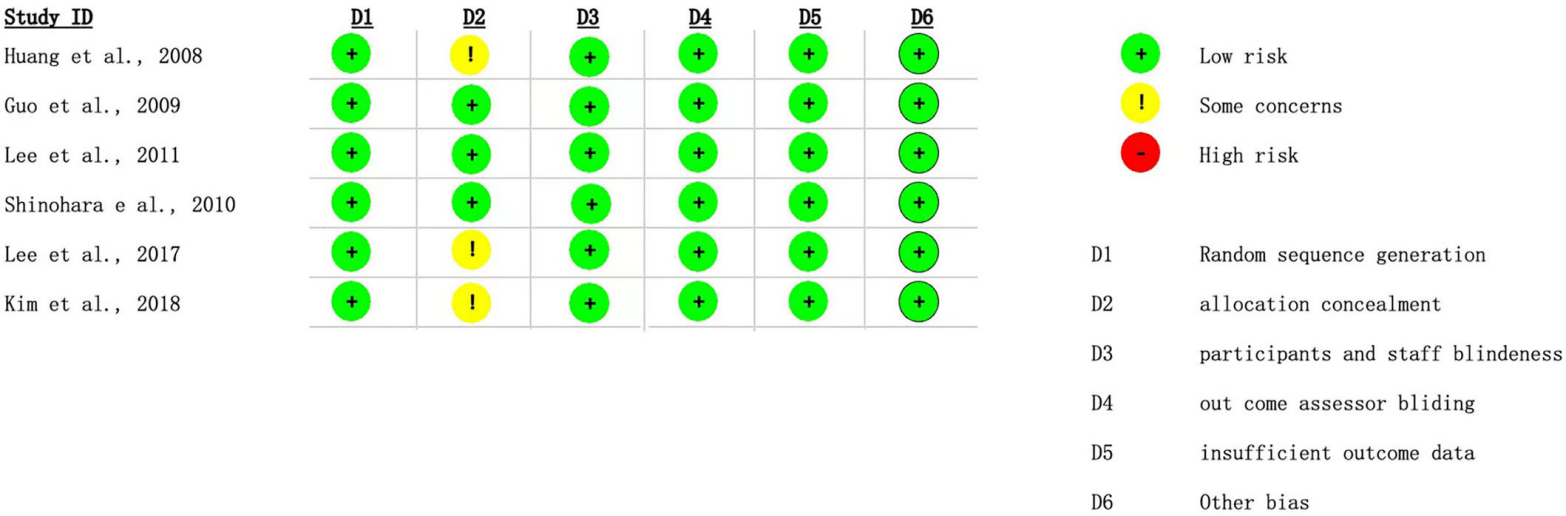
Bias assessment.

Two reviewers (EC and CL) independently evaluated the quality of the included randomized control trials (RCTs) using a modified version of the Cochrane risk of bias tool (RoB2) for randomized trials to address the risk of bias. Any disagreements between the rater of pieces of evidence are resolved by a third examiner (FT) (14). The researchers graded the evidence quality based on random sequence generation, allocation concealment, participant and staff blindness, outcome assessor blinding, missing outcome data (which rated as high risk of bias if missing data exceed 10%), and other biases. The findings were presented using the MAGICapp (15) (Figure 2).

### Quality of Evidence

The GRADE form was used to evaluate the quality of research (14). We graded the quality of research as high, moderate, low, or very poor for each outcome based on imprecision, inconsistency, indirectness, publication bias, and overall risk of bias.

### Statistical Analysis

For dichotomous results, the researchers used the Mantel–Haenszel method to measure overview risk ratios (RRs) and 95% CIs and used a random-effects model to account for the between-study heterogeneity. The researchers further used the Cochrane Q statistics and the I2 test to determine the heterogeneity of the included studies and used the RevMan 5.4 to conduct the meta-analysis.

## Results

### Study Identification and Trial Characteristics

Figure 1 presents the findings of the researchers' included studies. A total of six studies (4, 5, 7, 8, 16, 17) were included in the analysis with a total of 5,617 patients. All studies compared cilostazol monotherapy to aspirin monotherapy. The researchers found 2,524 documents in electronic libraries, 507 of which were duplicates and further reviewed 23 full-text articles with omissions on the 2017 records, depending on the title and abstract.

Table 1 summarizes the characteristics of the six included studies. Four included trials administered with cilostazol at 100 mg two times/day and aspirin at 100 mg/day. One of the trials administered cilostazol at a dose of 200 mg/day and aspirin at 300 mg/day. Another study included a trial administered with cilostazol at 200 mg/day and aspirin at 100 mg/day. Moreover, one included study administered cilostazol at a dose of 100 mg two times daily and aspirin at 81 mg/day. All studies were conducted in Asian countries as a result of cilostazol being mainly used in Asian countries. The patient demographics are summarized in Table 2.

**Table 1:** Characteristics of included studies.

**References**	**Design**	**Study period**	**Follow-up (months)**	**Medications**		**Total No**.	**Primary outcome**	**Cilostazol**	**Aspirin**	***P*-value**
				**Cilostazol**	**Aspirin**					
Huang et al. (5)	Multicenter Double-blind	May 2004–Dec. 2004	12–18	100 mg twice/day	100 mg/day	*N* = 720 • Ischemic within previous 1–6 months	Occurrence of stroke	0.28	0.85	0.18
Guo et al. (16)		May 2004–Dec. 2005	12	100 mg twice/day	100 twice/day	*N* = 68 • Ischemic within 1–6 months	Cerebro-vascular aggravation	1%	1%	0.90
Lee et al. (8)	Double blind Non-inferiority	Jan.2006–Mar. 2008	3	200 mg/day	300 mg/day	*N* = 458	mRS score of 0–2 at 90 days	173/231	165/227	0.90
Shinohara et al. (4)	Randomized Double-blind Non-inferiority	Dec. 2003–Oct. 2006	29^*^	100 mg twice/day	81 mg/day	*N* = 2,757 • Non-cardioem-bolic cerebral infarction previous 26 weeks	Recurrent stroke	82/1,337	113/1,335	0.036
Lee et al. (7)	Double-blind	March 2012–Oct. 2014	3	100 mg twice/day	100 mg/day	*N* = 80 • Acute ischemic stroke/TIA	Serious adverse events	2/40	5/40	0.235
Kim et al. (17)	Multicenter	Aug 2009–Aug 2015	22.8^**^	100 mg twice/day	100 mg/day	*N* = 1,534 • Non-cardioembolic ischemic stroke/TIA within 180 days	Composite of major vascular events	63/755	80/757	0.008

* *Mean follow-up*.

** *Median follow-up*.

**Table 2:** Included studies patient's demographics.

**Studies**	**Treatment**	**Age (years)**	**Male (%)**	HTN (%)*	DM (%)*	**DLP (%)**	**Smoker (%)**	Systolic BP^*^(mm Hg)	Diastolic BP^*^(mm Hg)	HLD (%)^*^
Huang et al. (5)	Cilostazol	60 ± 10	67	79	18	-	-	135 ± 17	83 ± 9	27
	Aspirin	60 ± 10	70	79	18	-	-	138 ± 18	83 ± 11	31
Guo et al. (16)	Cilostazol	59 ± 11	35	68	6	44	-	-	-	29
	Aspirin	62 ± 11	35	65	12	47	-	-	-	47
Lee et al. (8)	Cilostazol	63 ± 12	64	67	37	-	41	144 ± 25	84 ± 14	39
	Aspirin	63 ± 12	59	63	32	-	40	140 ± 22	82 ± 11	44
Shinohara et al. (4)	Cilostazol	64 ± 9	72	73	29	42	29	-	-	-
	Aspirin	63 ± 9	72	74	29	45	30	-	-	-
Lee et al. (7)	Cilostazol	54 ± 13	72	69	16	-	41	-	-	44
	Aspirin	60 ± 12	59	82	29	-	47	-	-	38
Kim et al. (17)	Cilostazol	66 ± 11	62	89	32	43	19	135 ± 18	80 ± 12	-
	Aspirin	66 ± 11	62	89	33	44	21	136 ± 18	80 ± 12	-

* *HTN, hypertension; DM, diabetes mellitus; DLP, dyslipidemia; BP, blood pressure; HLD, hyperlipidemia*.

### Risk of Bias

Figure 2 presented the risks of bias of the six included RCT studies. The appropriateness in estimating the effect of assignment to intervention is unclear in three RCTs. Otherwise, the overall risks of bias are low for the six included RCTs.

### Efficacy Outcomes

**Figure 3:**
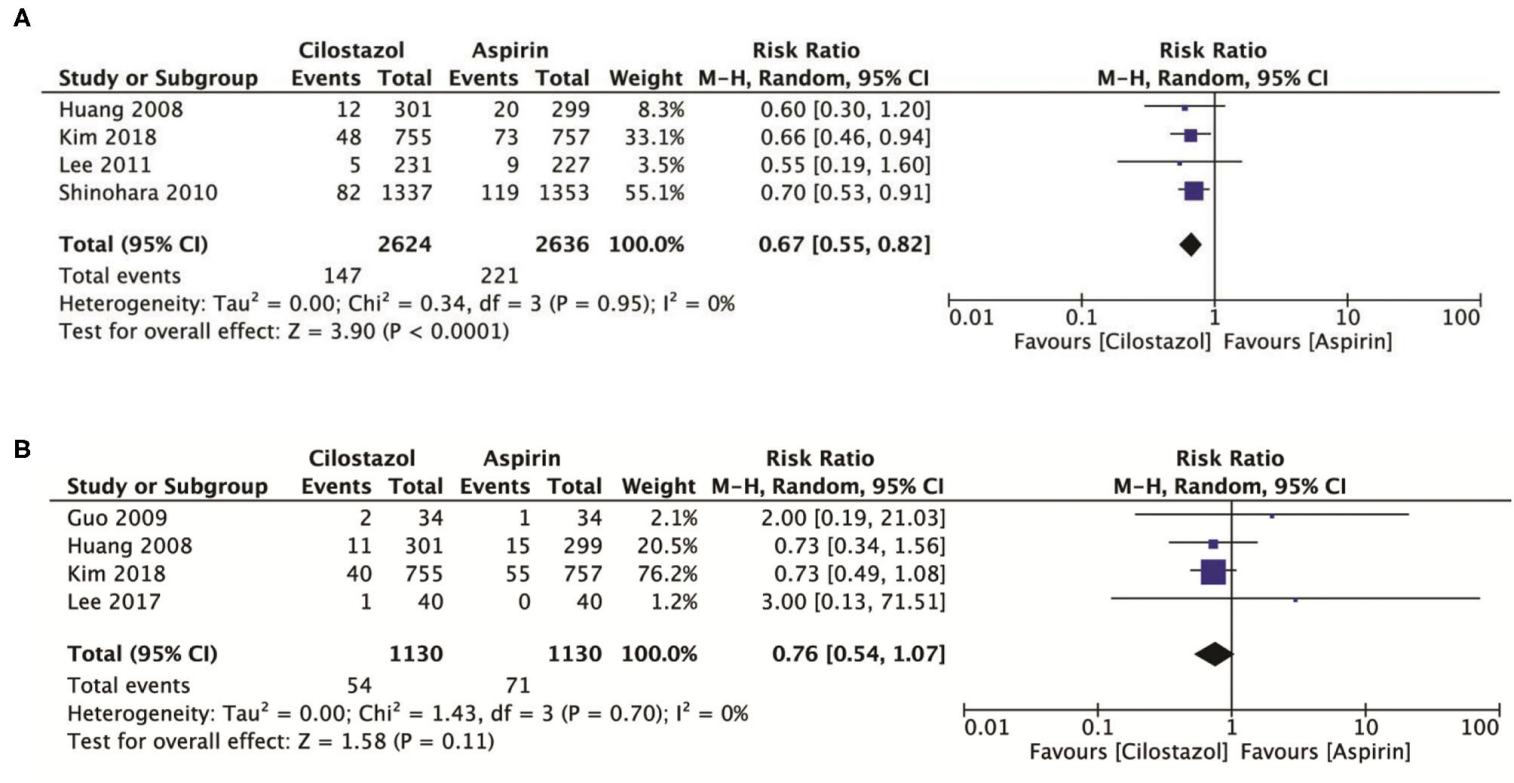
**(A)** A forest plot of comparison: any stroke; **(B)** A forest plot of comparison: ischemic stroke.

Compared with aspirin alone, a total of four studies with 5,260 patients showed that cilostazol monotherapy significantly reduced the risk of any stroke (RR: 0.67; 95% CI: 0.55–0.82, *p* < 0.0001) (Figure 3A). Four studies with 2,260 patients showed that cilostazol monotherapy was also associated with a lower ischemic stroke rate, however the results recorded were not significantly different (RR: 0.76; 95% CI: 0.54–1.07, *p* = 0.11) (Figure 3B).

### Safety Outcomes

**Figure 4:**
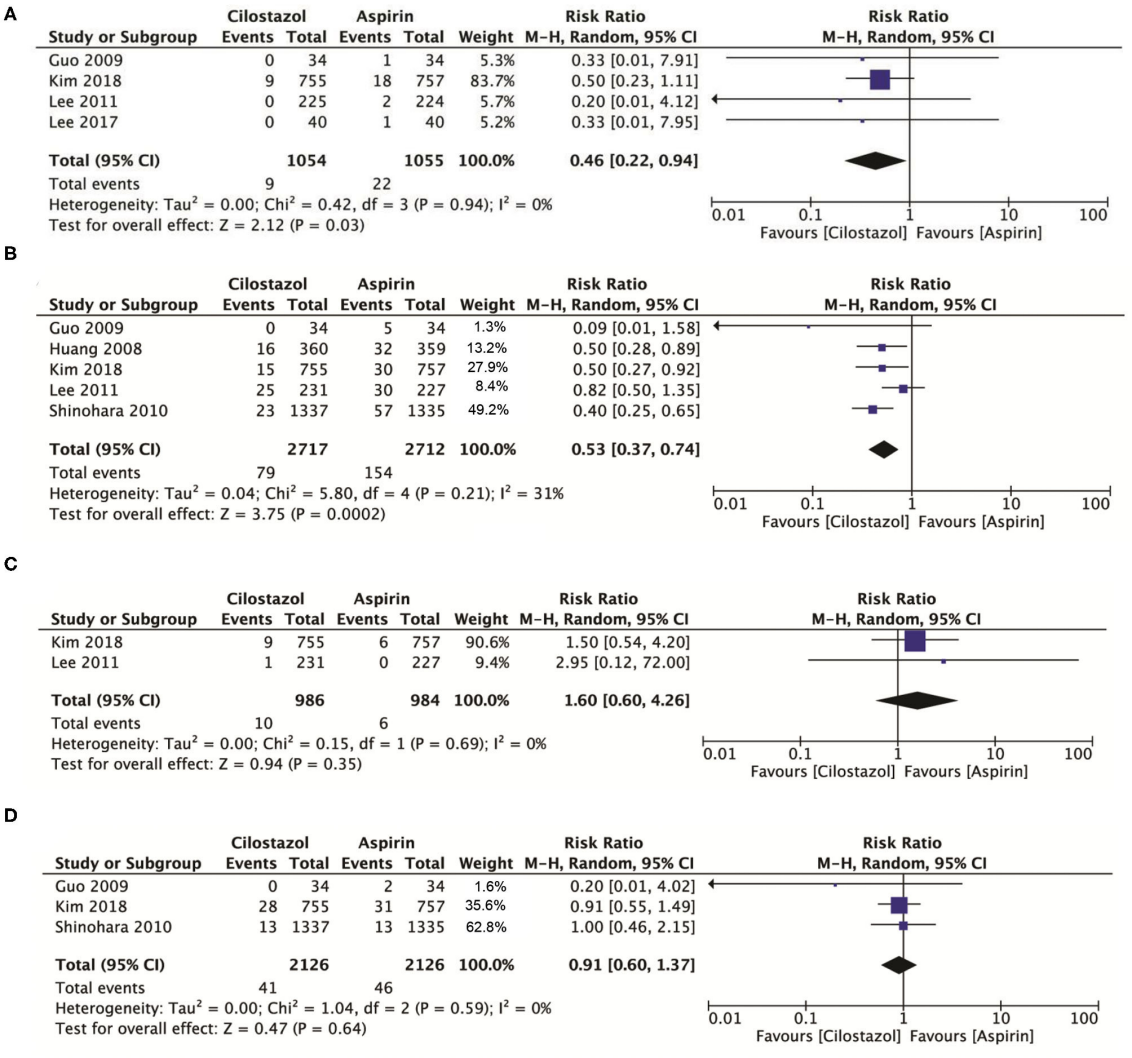
**(A)** A forest plot of comparison: intracranial bleeding; **(B)** A forest plot of comparison: any bleeding; **(C)** A forest plot of comparison: vascular death; **(D)** A forest plot of comparison: any death.

Compared with aspirin alone, a total of four studies with 2,109 patients showed that cilostazol monotherapy significantly reduced intracranial bleeding (RR: 0.46; 95% CI: 0.22–0.94, *p* = 0.03) (Figure 4A) and significantly reduced any bleeding risk (RR: 0.53; 95% CI: 0.37–0.74, *p* = 0.0002) (Figure 4B). However, there was no significant difference between cilostazol and aspirin alone for vascular death and all-cause mortality (RR: 1.60; 95% CI: 0.60–4.26 *p* = 0.35) (Figure 4C) (RR: 0.91; 95% CI: 0.60–1.37, *p* = 0.64) (Figure 4D).

### Adverse Outcomes

**Figure 5:**
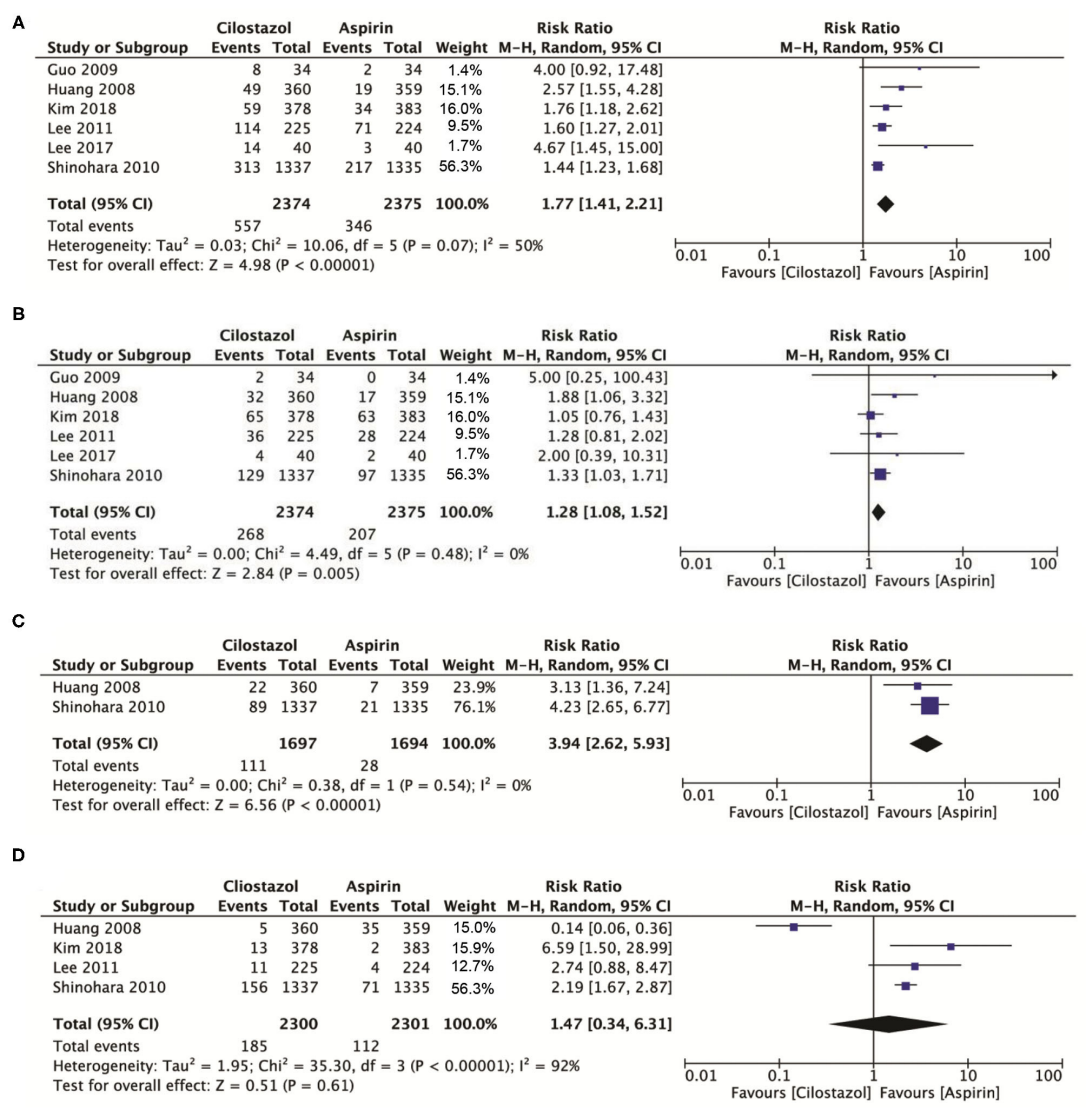
**(A)** A forest plot of comparison: headache; **(B)** A forest plot of comparison: dizziness; **(C)** A forest plot of comparison: tachycardia; **(D)** A forest plot of comparison: palpitation.

A total of six studies involving 4,740 patients showed that cilostazol was associated with a higher incidence of headache compared with aspirin monotherapy (RR: 1.77; 95% CI: 1.41–2.21, *p* < 0.00001) (Figure 5A), while cilostazol also significantly increased the frequency of dizziness (RR: 1.28; 95% CI: 1.08–1.52, *p* = 0.005) (Figure 5B). Two studies with 3,391 patients showed that cilostazol monotherapy significantly increased the tachycardia risk compared to aspirin monotherapy (RR: 3.94; 95% CI: 2.62–5.93, *p* < 0.00001) (Figure 5C). However, four studies with 4,601 patients showed that cilostazol did not significantly increase the palpitation frequency compared to aspirin monotherapy (RR: 1.47; 95% CI: 0.34–6.31, *p* = 0.61) (Figure 5D).

## Discussion

The researchers made some potentially valuable findings in this meta-analysis of six RCTs (*n* = 29,032) comparing cilostazol monotherapy to aspirin monotherapy. First, in patients with stroke, compared with aspirin, cilostazol significantly reduces any stroke incidence while reducing intracranial bleeding or any bleeding risks and does not significantly increase vascular death or death events. Second, although cilostazol alone is more efficient and safer than aspirin alone, cilostazol increases adverse events, primarily significantly increasing the incidence of headache, dizziness, and tachycardia. Similar results were found showing that the patients who received cilostazol had a 30% lower risk of persistent ischemic stroke, a 59% lower risk of intracranial hemorrhage, and a 29% lower risk of bleeding than patients who received aspirin (18). In addition, the current meta-analysis accounts for the adverse events in the results, which are the strength of this meta-analysis. Additionally, low heterogeneity (*I*^2^ = 0-31%) was observed in the evidence.

Stroke is the most common cause of disabilities and death (3). Despite the efforts of researchers and pharmaceutical companies, the risk of stroke recurrence remains high (19). The use of antiplatelet agents is recommended to reduce the long-term risk of non-cardioembolic ischemic stroke or TIA (20). Aspirin is a commonly used antiplatelet agent for secondary stroke prevention, but its benefit must be weighed against its bleeding risks, particularly in the aging population (20). It has been proved that aspirin is safe and beneficial in preventing stroke recurrence, but aspirin can only reduce recurrent vascular events by 20% (21). Previous meta-analyses have evaluated the effect of aspirin combined with clopidogrel on secondary stroke prevention, but, because of the high bleeding complications, no net benefit was found (22–28). Studies have recently found that it is more beneficial for acute high-risk patients treated with ticagrelor and aspirin than aspirin alone (29). Clearly, the optimal antiplatelet regimen, particularly in individuals at high risk for cerebral hemorrhages, such as those with a high burden of cerebral small-vessel disease, remains unclear and needs further investigation in well-designed clinical trials.

This study shows that Kim et al. did not find a significant effect of cilostazol and aspirin on intracranial hemorrhage, which may be due to their fragile small vessels and may lead to a greater incidence of intracranial bleeding (17). Similar findings were reported by Shinohara et al. (4); there was no significant intracranial hemorrhage between cilostazol and aspirin due to a high proportion of patients with a lacunar stroke in their study. Adverse events, including headaches and dizziness, occurred more frequently in the cilostazol group than in the aspirin group, but none were severe and all symptoms resolved after discontinuation or dose tapering of cilostazol. A study showed that some patients might avoid the adverse events caused by cilostazol by incremental increases in dose from 50 mg (4).

As a new type of antiplatelet inhibitor, cilostazol has anti-arterial thrombosis, prevents atherosclerosis, and improves vascular endothelial function (30–32). It can also regulate blood lipids and expand arterial blood vessels to stabilize plaques (33). It has a wide range of applications in treating peripheral vascular disease, preventing stent restenosis and thrombosis after PCI (34), and secondary prevention of ischemic stroke (16, 35). It is more suitable for aspirin-resistant or intolerant people, especially Asians (36).

Cilostazol is a selective inhibitor of phosphodiesterase, which increases intracellular activity, thereby inhibiting platelet aggregation (37–39). In some respects, the drug is a potent drug that can replace aspirin. For example, in previous clinical trials and meta-analyses, cilostazol significantly reduced the risk of stroke recurrence and lower bleeding events compared to aspirin (40, 41). Moreover, our current meta-analysis is in line with previous meta-analyses that found cilostazol to be more beneficial in patients with ischemic stroke (18, 42).

Although current meta-analysis and previous studies have shown the effectiveness and relative safety of using cilostazol as secondary prevention of stroke, research also shows that, even in non-Asian populations, cilostazol may have a significant potential for secondary stroke prevention. Patients with bleeding tendencies, such as small vessel disease and numerous microbleeds, or those who have hemorrhagic strokes, are likely to benefit from cilostazol treatment (43). However, compared with Asians, cilostazol is relatively uncommon in Western populations. Several reasons may explain this uncommonness. First, intracranial atherosclerosis (ICAS) is the leading cause of stroke, and Asians more often have ICAS than Caucasians (44). Second, the absorption, metabolism, and excretion of cilostazol may be modified by race/ethnicity (45). For instance, common polymorphisms in the CYP2C19 gene for clopidogrel metabolism vary by race/ethnicity, noted in ~30% of Caucasians, 40% of blacks, and more than 50% of East Asians (46). The pharmacogenetic of cilostazol is less well described, but it has been observed that genetic polymorphisms in CYP2C19 genes influence cilostazol pharmacokinetics (47). This is, therefore, possible that race/ethnicity may influence the effect of cilostazol on lowering ischemic stroke, ICH, and bleeding in non-Asian populations, but more studies are needed to examine how genetics and environment may affect the metabolism of cilostazol (18). Third, due to the lack of sufficient RCTs to study the effectiveness and safety of cilostazol as secondary prevention of stroke in Western populations, non-Asian physicians are not inclined to use cilostazol (44). Therefore, further pieces of research on the effect of cilostazol on different groups of people and ethnicity are needed.

The current meta-analysis has several limitations. First, the patients included in the studies were mainly from the Asian region, which will lead to regional deviations in the results. Large-scale research is required to determine whether the researchers' results are valid and similar in non-Asian populations. Second, the present meta-analysis did not conduct subgroup analysis to assess the impact of time to randomization following a stroke and the length of time spent taking the research drug on effectiveness and safety outcomes. Also, sensitivity analysis was not performed. Third, the follow-up length is different, ranging from 3 months to 29 months. Finally, in MI, there was inter-study variability in the outcomes. Such inherent variations between the researchers' included trials, such as sample demographics, non-cardioembolic infarction inclusion/exclusion requirements, stroke occurrence, treatment, follow-up duration, drug compliance rates, and other factors, are not considered.

## Conclusions

Cilostazol is more effective than aspirin alone in reducing the recurrence rate of stroke without increasing the risk of bleeding and death. However, when using cilostazol, the significantly increased probability of adverse events cannot be ignored.

## Data Availability Statement

The original contributions presented in the study are included in the article/supplementary material, further inquiries can be directed to the corresponding author/s.

## Author Contributions

EC, JC, CL, XZ, ZF, and FT conceived and designed the study. JC, SY, KZ, and WL selected the studies and collected the data. EC, JC, ZX, YZ, and FT analyzed the data. EC and FT drafted and revised the article. All authors interpreted the results, read, and approved the final version of the manuscript.

## Funding

This study was supported by the Key Laboratory of Cerebrovascular Disease of Gansu Province (No. 20JR10RA431), Innovation and Entrepreneurship Talent Project of Lanzhou (No. 2017-RC-57), National Natural Science Foundation of China (Nos. 81870329 and 81960673), Natural Science Foundation of Gansu Province (21JR1RA135), Cuiying Technological Innovation Foundation of Lanzhou University Second Hospital (CY2019-MS03), and Industrial Support Program for Colleges and Universities in Gansu Province (2020C-04).

## Conflict of Interest

The authors declare that the research was conducted in the absence of any commercial or financial relationships that could be construed as a potential conflict of interest.

## Publisher's Note

All claims expressed in this article are solely those of the authors and do not necessarily represent those of their affiliated organizations, or those of the publisher, the editors and the reviewers. Any product that may be evaluated in this article, or claim that may be made by its manufacturer, is not guaranteed or endorsed by the publisher.
